# Polymorphisms in K13 and Falcipain-2 Associated with Artemisinin Resistance Are Not Prevalent in *Plasmodium falciparum* Isolated from Ugandan Children

**DOI:** 10.1371/journal.pone.0105690

**Published:** 2014-08-21

**Authors:** Melissa D. Conrad, Victor Bigira, James Kapisi, Mary Muhindo, Moses R. Kamya, Diane V. Havlir, Grant Dorsey, Philip J. Rosenthal

**Affiliations:** 1 Department of Medicine, University of California San Francisco, San Francisco, California, United States of America; 2 Infectious Diseases Research Collaboration, Kampala, Uganda; 3 Department of Medicine, Makerere University College of Health Sciences, Kampala, Uganda; Ehime University, Japan

## Abstract

The emergence of resistance to artemisinin derivatives in Southeast Asia, manifested as delayed clearance of *Plasmodium falciparum* following treatment with artemisinins, is a major concern. Recently, the artemisinin resistance phenotype was attributed to mutations in portions of a *P. falciparum* gene (*PF3D7_1343700*) encoding kelch (K13) propeller domains, providing a molecular marker to monitor the spread of resistance. The *P. falciparum* cysteine protease falcipain-2 (FP2; *PF3D7_1115700*) has been shown to contribute to artemisinin action, as hemoglobin degradation is required for potent drug activity, and a stop mutation in the FP2 gene was identified in parasites selected for artemisinin resistance. Although delayed parasite clearance after artemisinin-based combination therapy (ACT) has not yet been noted in Uganda and ACTs remain highly efficacious, characterizing the diversity of these genes is important to assess the potential for resistance selection and to provide a baseline for future surveillance. We therefore sequenced the K13-propeller domain and FP2 gene in *P. falciparum* isolates from children previously treated with ACT in Uganda, including samples from 2006–7 (n = 49) and from 2010–12 (n = 175). Using 3D7 as the reference genome, we identified 5 non-synonymous polymorphisms in the K13-propeller domain (133 isolates) and 35 in FP2 (160 isolates); these did not include the polymorphisms recently associated with resistance after *in vitro* selection or identified in isolates from Asia. The prevalence of K13-propeller and FP2 polymorphisms did not increase over time, and was not associated with either time since prior receipt of an ACT or the persistence of parasites ≥2 days following treatment with an ACT. Thus, the K13-propeller and FP2 polymorphisms associated with artemisinin resistance are not prevalent in Uganda, and we did not see evidence for selection of polymorphisms in these genes.

## Introduction

The emergence of resistance to artemisinin and its derivatives in Southeast Asia, manifested as delayed clearance of *Plasmodium falciparum* following treatment with these drugs, is of great concern [1]–[3]. As the first-line treatment for falciparum malaria in nearly all endemic countries, artemisinin-based combination therapies (ACTs) are of critical importance, and the spread of resistance may be catastrophic for malaria control and elimination efforts around the globe. Previous experience with the spread of choloroquine- and sulfadoxine/pyrimethamine-resistant parasites from Asia to Africa demonstrates that the spread of artemisinin resistance is likely, and that vigilant surveillance for resistant parasites is warranted [4], [5].

Currently, multiple ACTs remain very effective for the treatment of malaria in Africa, as demonstrated by rapid parasite clearance and low rates of recrudescence after therapy in clinical trials, and good *ex vivo* sensitivity of clinical isolates [6]–[10]. However, recent changes in drug policy in East Africa, with artemether-lumefantrine (AL) established as the first line treatment for uncomplicated malaria, have been accompanied by the selection of polymorphisms associated with decreased lumefantrine sensitivity [11], [12] and by decreased *ex vivo* lumefantrine sensitivity [13]. Reduced sensitivity to ACT partner drugs may exacerbate selection of artemisinin resistance, jeopardizing our most important antimalarial therapies.

Recent work attributed the artemisinin resistance phenotype found in Southeast Asia to mutations in *PF3D7_1343700,* which encodes a protein (K13) homologous to kelch proteins from other organisms [14]. Parasites selected for resistance to artemisinin demonstrated multiple mutations predicted to be in propeller domains of the protein. The mutations were associated with improved parasite survival after pulses of dihydroartemisinin, an *in vitro* correlate of artemisinin resistance [15], and with delayed clearance after artemisinin therapy in Cambodia [14]. Specifically, the M476I mutation was selected *in vitro* in a Tanzanian parasite by longstanding cyclic artemisinin pressure, and 3 additional polymorphisms prevalent in Cambodian field isolates (C580Y, R539T, and Y493H) were associated with delayed clearance after therapy.

The cysteine protease falcipain-2 (FP2; *PF3D7_1115700*) is a principal *P. falciparum* hemoglobinase [16]. Inhibition of this protease or knockout of the gene blocked hemoglobin hydrolysis in trophozoites [17] and led to decreased artemisinin activity, as hemoglobin is required for a potent antimalarial effect [18]. Interestingly, parasites selected *in vitro* for artemisinin resistance had a nonsense mutation at codon 69 of the FP2 gene [14], suggesting that parasites partially blocked hemoglobin processing to limit toxicity from artemisinin.

Although ACT remains highly efficacious for the treatment of falciparum malaria and delayed parasite clearance after ACT has not been noted in Uganda, it was important to characterize the diversity of genes in which polymorphisms may contribute to artemisinin resistance. Our goals were to characterize the diversity of the K13 and FP2 genes and to determine if artemisinin selective pressure or relative delays in parasite clearance after therapy were associated with particular genotypes. We therefore sequenced these genes in *P. falciparum* isolates collected from Ugandan children under varied selective pressure from recent therapy with ACTs.

## Materials and Methods

### Selection of *P. falciparum* isolates

Isolates from children not considered under artemisinin pressure were from clinical trials in Apac [19] and Kanungu [20] performed in 2006–7 (n = 49). Samples were collected for Giemsa-stained blood smears and blood spots on filter paper before treatment, and since use of ACTs outside of trials was very uncommon in Uganda in this period, artemisinin pressure on infecting parasites was expected to be minimal. Newer samples (n = 175) were collected in 2010–12 from a clinical trial in Tororo in which children were randomly assigned chemoprevention with daily trimethoprim-sulfamethoxazole, monthly sulfadoxine-pyrimethamine, monthly dihydroartemisinin/piperaquine (DP) or no chemoprevention, beginning at age 6 months [21] (ClinicalTrials.gov Identifier NCT00948896). In this trial participants received all of their medical care at a designated study clinic, where Giemsa-stained thick blood smears and blood spots on filter paper were obtained monthly and in any children who presented with a documented fever or history of fever in the previous 24 hours. All episodes of uncomplicated malaria (fever with any level of parasitemia) diagnosed during the trial were treated with AL. From the newer trial, 175 samples were selected for genotyping at K13 and FP2, 141 of these considered under potential artemisinin pressure, 93 from individuals in the monthly DP treatment arm, and 48 in other arms, but treated for a prior episode of malaria with AL within 30 days prior to collection. The remaining 34 isolates from the newer trial, all from December 2012, were not under drug pressure, but were sequenced to increase representation of new samples. Ethical approval was obtained for all trials from the Uganda National Council for Science and Technology, the Makerere University School of Medicine Research and Ethics Committee, and the University of California, San Francisco Committee on Human Research, and written informed consent was obtained from caregivers of participants.

### Analysis of *P. falciparum* isolates

DNA was extracted from blood spots into 100 µl of water using Chelex-100 (Bio-Rad). For the K13 gene, we used (hemi)nested-PCR to amplify a 1282 bp fragment extending from nucleotides 828 to 2110. For the FP2 gene, 2 nested PCR reactions were utilized to amplify the entire gene, spanning nucleotides 1–873 and 271–1455. For both loci, first and second round amplifications were performed in 25 µl reactions containing 160 nM primers (Integrated DNA Technologies), 160 µM dNTPs (Invitrogen), 1 unit Taq DNA Polymerase (New England BioLabs), and 2 µl DNA/primary reaction template in 1x Standard Taq Buffer (New England BioLabs). Primers sequences were: K13 primary reaction (K13-1: CGGAGTGACCAAATCTGGGA/K13-4: GGGAATCTGGTGGTAACAGC) [14]; K13 nested reaction (KelchNF: TTGAAGAACAGAAATTACATGATGA/K13–1); FP2 primary reaction (FP2F: TGTAGCAAGAACGTTTTGTGTAAAT/FP2R: GGTAAAGGAAAAATTAGTAAGGATGC); FP2 nested reaction 1 (FP2NF: TGTGTAAATTAAAGATAAAAGTGCAAA/FP2intR: GCATATTGTGATTCTACGGAACC); and FP2 nested reaction 2 (FP2intF: AAAAAGCCCTAATGGCAAGAA/FP2NR: GGTCCCTTTTTAAAATACTATTGACA). Thermocycling conditions were 94°C×5 min; 35 cycles of 94°C×30 sec, 60°C×60 sec, 72°C×90 sec; 72°C×10 min for all reactions. Products were electrophoresed on 1.5% agarose gels, remnant primers and nucleotides were removed using ExoSap IT (Affymetrix), and samples were bi-directionally sequenced using ABI BigDye Terminator chemistry at the UCSF Genomics Core Facility. Reads were aligned to reference PF3D7_1343700 (www.plasmodb.org; version 11.0) and manually scored for polymorphisms using Sequencher v. 5.2.4 (Gene Codes Corporation). Sequences were deposited in GenBank under accession numbers KM187656–KM188048.

### Statistics and Analyses

Data analysis was with Stata version 12 (Stata Corp). Outcomes of interest were the prevalence of mutant alleles for each polymorphic locus. Exposure variables of interest were persistence of parasitemia <2 days or ≥2 days after the onset of treatment, if samples were isolated within 30 days of previous ACT or not, and whether isolates were collected in 2006–2007 or in 2012. Significance was determined using Fisher’s exact test. To determine if the genetic diversity of FP2 changed between 2006–7 and 2012 we performed exact tests of sample differentiation using Arlequin v. 3.5. In all analyses, a two-tailed P value <0.05 was considered significant.

## Results

We studied samples from 222 malaria episodes that occurred in 2006–7 and 2010–12 under a range of selective pressure from ACTs. All episodes were *P. falciparum* monoinfections, except for one from 2012, which was determined by microscopy to contain *P. falciparum* and *P. ovale*. For samples from 2010–12, clearance of parasites after treatment with AL was generally prompt; parasitemia was present in only 13/175 patients ≥2 days after the initiation of treatment.

We sequenced the gene encoding the K13-propeller domain in *P. falciparum* isolated from 133 episodes diagnosed from 2010–12. Compared to the reference 3D7 strain, we observed 5 non-synonymous polymorphisms (Table 1), 2 in K13-propeller blade 5, and 1 each in blades 1, 3 and 4 (based on homology with the human KEAP1, KLHL12 and KLH2 proteins [14]). All polymorphisms were uncommon, and each was present in only one isolate. Only two polymorphisms, I465T and L619S, were found in the same sample, which was a mixed-species infection, containing both *P. falciparum* and *P. ovale* parasites. Four of the five non-synonymous polymorphisms were found in isolates also containing the wild type allele; only the V637D mutation was found as a pure mutant genotype. Notably, we did not observe the C580Y, R539T, or Y493H substitutions that were associated with delayed parasite clearance in Southeast Asian parasites, nor the M476I mutation that was selected *in vitro* with artemisinin pressure. In addition to the K13-propeller domain, we sequenced the upstream region of the gene, starting at codon 159, in a subset of 29 samples. Within this region we identified an additional 3 non-synonymous SNPs (Table 1). Polymorphism at one locus in this region (K189) was seen in 34% of isolates.

**Table 1:** *P. falciparum* K13-propeller amino acid and nucleotide substitutions observed in Tororo, Uganda.

Codon	Propellerblade	Type	Referenceaminoacid	Mutantaminoacid	NucleotideLocus	Referenceallele	Mutantallele	n/N
189^a^	-	NS	K	T	566	A	C	10/29
189^a^	-	NS	K	N	567	A	T	1/29
334	-	NS	F	L	1000	T	C	2/102
368^a^	-	Syn	L	L	1104	A	G	1/101^c^
439	-	Syn	F	F	1317	T	C	1/129
465	1	NS	I	T	1394	T	C	1/129
467	1	Syn	Q	Q	1401	A	G	1/131
522	2	Syn	S	S^b^	1566	T	C	1/132
558	3	NS	Y	H	1672	T	C	1/133
578^a^	4	NS	A	S	1732	G	T	1/133
617	5	NS	A	T	1849	G	A	1/133
619	5	NS	L	S	1856	T	C	1/133^c^
637	5	NS	V	D^b^	1910	T	A	1/132

All data are relative to reference sequence *PF3D7_1343700.*

n = number of samples containing mutant allele.

N = number of samples sequenced at locus.

a SNP has been previously identified (MalariaGen).

b Different SNP in same codon has been previously reported (MalariaGen).

c Polymorphism identified in mixed *P. falciparum/P. ovale* infection.

We sequenced the FP2 gene from 160 isolates collected in 2010–12, observing (compared to the 3D7 strain) 35 non-synonymous SNPs, 26 in the protease pro and folding domains and 9 in the catalytic domain, and also one deletion in the pro domain [16] (Table 2). These polymorphisms did not include the S69 stop mutation identified after *in vitro* selection for artemisinin resistance, but did include all 11 SNPs represented in 49 culture-adapted Cambodian isolates, but not associated with survival after pulses of dihydroartemisinin [14] (Table 2). The diversity identified in FP2 from clinical isolates was greater than that seen in surveys of *P. falciparum* laboratory strains. To determine if diversification of FP2 was associated with increasing use of ACTs, we sequenced 49 isolates collected from Ugandan children in 2006–07, pre-dating the widespread use of ACTs in Uganda. In these samples we identified 12 non-synonymous substitutions, 8 also seen in samples from 2012. We compared the prevalence of the mutant alleles for each locus in samples from 2006–7 (n = 49) and 2012 (n = 112), and found no significant differences (P for all comparisons ≥0.23). We also tested for population differentiation (F_st_) and found no significant differences between the two time periods (P = 0.99).

**Table 2:** *P. falciparum* FP2 non-synonymous amino acid and nucleotide substitutions observed in Tororo, Uganda.

Codon	ProteinDomain^f^	Ref. AA	Mut. AA	Nuc.Locus	Ref.Codon	Mut.Codon	n/N(2010–12)	n/N(2006–07)	n/N(2012)
4^a^^,^ b	Pre-	N	H	10	AAC	CAC	8/73	-	-
8^a^^,^ b	Pre-	A	I^c^	22–24	GCT	ATT	7/76	-	-
10^b^	Pre-	H	N	28	CAT	AAT	8/78	-	-
15^a^^,^ b ^,^ d	Pre-	Q	H	45	CAA	CAT	9/80	-	-
51^b^^,^ d	Transmem.^e^	V	I	151	GTT	ATT	14/90	-	-
55	Transmem.	F	I	163	TTT	ATT	1/90	-	-
59^b^^,^ d	Pro-	S	F	176	TCT	TTT	6/91	-	-
105^b^	Pro-	S	N	314	AGT	AAT	2/152	0/22	0/107
107^a^^,^ b	Pro-	K	M	320	AAG	ATG	1/158	0/26	0/111
108^a^^,^ b	Pro-	N	K	324	AAT	AAA	1/158	0/28	0/111
113^a^	Pro-	Y	N	337	TAC	AAC	1/158	0/33	0/111
115–122	Pro-	NEGNNNNNA	NNNNTLSD	342–369	-	-	1/158	0/33	0/111
134^b^	Pro-	T	K	401	ACA	AAA	1/160	0/41	0/112
161	Pro-	H	R^c^	482	CAT	CGT	1/160	0/44	0/112
169	Pro-	I	M	507	ATT	ATG	1/160	0/45	1/112
173^b^	Pro-	N	K	519	AAT	AAA	1/160	0/45	0/112
197^b^^,^ d	Pro-	N	K	591	AAT	AAA	1/160	0/49	1/112
204^b^^,^ d	Pro-	N	K	612	AAT	AAG	1/160	0/49	1/112
210	Pro-	E	Q	628	GAA	CAA	0/160	1/49	0/112
228^b^^,^ d	Pro-	S	T	683	AGT	ACT	16/160	5/49	13/112
245^a^	Pro-	M	I	735	ATG	ATA	1/160	0/49	1/112
248^a^	Folding	E	D	744	GAA	GAC	1/160	0/49	1/112
249	Folding	E	A	746	GAA	GCA	1/160	0/49	1/112
249	Folding	E	D	747	GAA	GAC	1/160	0/49	1/112
255^b^	Folding	K	G^c^	763–764	AAA	GGA	6/160	2/49	4/112
255^b^^,^ d	Folding	K	R^c^	764	AAA	AGA	152/160	46/49	106/112
257^b^^,^ d	Folding	N	E	769–771	AAT	GAA	158/160	48/49	110/112
299^b^	Catalytic	A	V	896	GCT	GTT	0/160	1/49	0/112
310	Catalytic	E	D	930	GAA	GAC	1/160	0/49	0/112
335	Catalytic	M	I	1005	ATG	ATT	2/160	1/49	2/112
337	Catalytic	E	D	1011	GAA	GAT	1/160	0/49	1/112
343^b^^,^ d	Catalytic	T	P	1027	ACA	CCA	156/160	47/49	110/112
345^b^^,^ d	Catalytic	D	G	1034	GAT	GGT	157/260	47/49	111/112
365	Catalytic	K	T	1094	AAA	ACA	1/158	0/49	1/110
384^b^	Catalytic	R	S	1152	AGA	AGC	0/158	1/49	0/110
393	Catalytic	V	I	1177	GTA	ATA	1/158	0/49	0/110
398	Catalytic	D	V	1193	GAT	GTT	0/158	1/49	0/110
414^b^^,^ d	Catalytic	Q	E	1240	CAA	GAA	41/157	15/47	27/109
414	Catalytic	Q	L	1241	CAA	CTA	1/157	0/47	1/109

All data are relative to reference sequence *PF3D7_1343700.*

n = number of samples containing mutant allele.

N = number of samples sequenced at locus.

a SNP reported in MalariaGen.

b SNP reported in PlasmoDB, v. 11.0.

c Different SNP in same codon has been previously reported (MalariaGen or PlasmoDB).

d SNP reported to be present in culture adapted Cambodian strains by Ariey *et al*.[14].

e Transmembrane boundaries as indicated in PlasmoDB v. 11.0 (amino acids 35–57).

To determine if mutations were associated with recent ACT use, we compared the prevalence of K13 and FP2 mutations in isolates collected within 30 days (K13: n = 104; FP2: n = 132) or >60 days (K13: n = 18; FP2: n = 18) after a prior treatment. We found no significant differences at any locus (P≥0.15 for K13 and ≥0.11 for FP2). We also compared sequences in isolates from malaria episodes in which parasites were detected in blood smears ≥2 days after the onset of treatment (K13: n = 9; FP2: n = 12) to those from episodes in which parasites cleared within 2 days; no differences in SNP prevalence were seen (P≥0.58 for K13 and ≥0.06 for FP2). Thus, neither recent prior use of an ACT, nor somewhat delayed parasite clearance was associated with the presence of K13 or FP2 SNPs in Ugandan isolates.

## Discussion

Artemisinin resistance, manifested as delayed parasite clearance [1] and correlated with diminished action of pulses of artemisinins *in vitro* [15], has recently been identified in Southeast Asia and associated with mutations in the regions of the *P. falciparum* K13 gene that encode the propeller domains [9]. To characterize potential resistance markers in isolates from Uganda, we surveyed K13-propeller polymorphisms in recent isolates under varied levels of selective pressure due to prior therapy with ACTs. We identified limited diversity within the K13 gene, and did not detect any of the polymorphisms associated with artemisinin resistance in Southeast Asia. In addition, we found that the prevalences of K13-propeller polymorphisms identified in Uganda were not associated with recent use of ACTs or with the persistence of parasites ≥2 days following treatment with ACTs.

Prior studies demonstrated that parasites treated with a FP2 inhibitor and FP2 deletion mutants were protected against an artemisinin pulse *in vitro*, indicating that the hemoglobinase FP2 is necessary for optimal activity of artemisinins [18]. Interestingly, parasites selected *in vitro* for artemisinin resistance contained a stop mutation in the FP2 gene [14]. To evaluate FP2 polymorphisms in Uganda over time, we sequenced the FP2 gene in isolates collected before and after the widespread implementation of ACT in Uganda. We identified notable sequence diversity, but did not detect the mutation selected by *in vitro* artemisinin pressure [14]. In addition, diversity in the FP2 sequence did not change over time and was not influenced by recent ACT use.

Our analysis had limitations. First, with the short half-life of artemisinins, the primary selective pressure is likely that of the ACT partner drug, rather than the artemisinin component, so studying isolates from recently treated subjects may not be a sensitive means of identifying rare artemisinin-resistant parasites [22]. Second, as delayed clearance of parasites after therapy is uncommon and associated with high baseline parasitemia in Uganda, persistent parasitemia 2 days after the onset of therapy is likely not a reliable indicator of resistance [6], [8]. Third, since the best means of identifying rare resistant parasites is uncertain, parasites with a range of selective pressures were chosen for sequencing, limiting statistical power for some comparisons. Finally, due to the lack of sensitivity of Sanger sequencing in detecting minority alleles in mixed infections, we may have missed low abundance alleles which might play a role in artemisinin resistance. However, because of the scarcity of the artemisinin-resistance phenotype in Uganda, we felt that it was more useful to study parasites under potential selection or with somewhat slow clearance, rather than to survey parasites collected randomly.

The absence of known molecular markers of artemisinin resistance in studied isolates is consistent with clinical findings, as ACTs remain highly efficacious in Uganda, where delayed parasite clearance following treatment with ACTs has been uncommon [6]–[9]. These results are encouraging, and suggest that artemisinin resistance is not yet established in Uganda. However, continued drug pressure, facilitated by decreasing sensitivity to ACT partner drugs, will offer strong selection for resistance, either driving the spread of resistant parasites imported from Asia, or selecting for *de novo* evolution of resistance (possibly via different mechanisms than in Asia) in Africa. Thus, continued studies to better characterize the genetics of artemisinin resistance and continued surveillance for markers of resistance in Africa are urgent priorities.
